# Enterohemorrhagic Escherichia coli Colonization of Human Colonic Epithelium *In Vitro* and *Ex Vivo*

**DOI:** 10.1128/IAI.02928-14

**Published:** 2015-02-13

**Authors:** Steven B. Lewis, Vivienne Cook, Richard Tighe, Stephanie Schüller

**Affiliations:** a Norwich Medical School, University of East Anglia, Norwich, United Kingdom; b Gut Health and Food Safety Programme, Institute of Food Research, Norwich, United Kingdom; c Gastroenterology Department, Norfolk and Norwich University Hospital, Norwich, United Kingdom

## Abstract

Enterohemorrhagic Escherichia coli (EHEC) is an important foodborne pathogen causing gastroenteritis and more severe complications, such as hemorrhagic colitis and hemolytic uremic syndrome. Pathology is most pronounced in the colon, but to date there is no direct clinical evidence showing EHEC binding to the colonic epithelium in patients. In this study, we investigated EHEC adherence to the human colon by using *in vitro* organ culture (IVOC) of colonic biopsy samples and polarized T84 colon carcinoma cells. We show for the first time that EHEC colonizes human colonic biopsy samples by forming typical attaching and effacing (A/E) lesions which are dependent on EHEC type III secretion (T3S) and binding of the outer membrane protein intimin to the translocated intimin receptor (Tir). A/E lesion formation was dependent on oxygen levels and suppressed under oxygen-rich culture conditions routinely used for IVOC. In contrast, EHEC adherence to polarized T84 cells occurred independently of T3S and intimin and did not involve Tir translocation into the host cell membrane. Colonization of neither biopsy samples nor T84 cells was significantly affected by expression of Shiga toxins. Our study suggests that EHEC colonizes and forms stable A/E lesions on the human colon, which are likely to contribute to intestinal pathology during infection. Furthermore, care needs to be taken when using cell culture models, as they might not reflect the *in vivo* situation.

## INTRODUCTION

Enterohemorrhagic Escherichia coli (EHEC) is a major cause of bacterial diarrhea in the developed world, and infections can lead to acute gastroenteritis, hemorrhagic colitis (HC), and systemic hemolytic uremic syndrome (HUS) (1–3). HC and HUS are associated with the release of bacterial Shiga toxins (Stxs), which primarily affect the kidneys and central nervous system, which express large amounts of the Stx glycolipid receptor globotriaosylceramide (Gb3) (4, 5). In contrast, the development of diarrhea is linked to a type III secretion system (T3SS), which enables the bacteria to colonize human intestinal epithelium and modulate host cell signal transduction by injecting bacterial effector proteins (6, 7). Initial events of type III secretion (T3S) comprise the formation of the EspA translocation tube and delivery of the translocated intimin receptor (Tir) into the host cell membrane (8, 9). This is followed by binding of the bacterial outer membrane adhesin intimin to Tir, which initiates formation of attaching and effacing (A/E) lesions (10). EHEC A/E lesion formation has been demonstrated in cultured cell lines and some animal models and is characterized by intimate attachment, microvillous effacement, and actin polymerization beneath adherent bacteria (11–14). Whereas microscopy has demonstrated adherent EHEC in the small intestine and the colon of gnotobiotic piglets, neonatal calves, and infant rabbits (12–14), similar direct evidence of EHEC binding to human colonic epithelium is lacking (15). This is surprising, as EHEC predominantly causes a colonic pathology in humans (15, 16), but the limited numbers of biopsy samples available in the early stages of EHEC disease, before the occurrence of extensive tissue damage, no doubt contribute to the lack of such evidence. *In vitro* organ culture (IVOC) of human endoscopic biopsy samples has been employed to investigate EHEC adherence, and these studies using Stx-negative EHEC strains and oxygen-rich culture conditions have demonstrated A/E lesion formation on the terminal ileum but not the colon (17, 18).

In the present study, we have reexamined EHEC adherence to colonic epithelium using EHEC wild-type strains and atmospheric oxygen levels (i.e., 20% atmospheric pressure). As it has previously been shown that Stxs promote EHEC adherence to HeLa cells and intestinal colonization in mice (19), we sought to determine whether Stx expression would also enable EHEC binding to human colonic epithelium. In addition, IVOC experiments are usually performed under oxygen-rich culture conditions (95% atmospheric pressure) to allow oxygen penetration into deeper tissues, but our earlier studies have demonstrated that oxygen inhibits EHEC T3S and A/E lesion formation (20), which might explain the lack of colonic adherence observed in previous IVOC studies. In addition to investigating EHEC adherence to human colonic explants, we have also included T84 human colon carcinoma cells, which are widely used as an *in vitro* model for colonic EHEC infection.

## MATERIALS AND METHODS

### Bacterial strains and culture conditions.

The bacterial strains used in this study are listed in Table 1. Bacteria were grown while they were standing in LB broth overnight at 37°C. Deletion mutants (except EDL933 Δ*espA*) were selected with kanamycin (50 μg/ml). Bacteria were spun down before infection and suspended in serum-free culture medium.

**TABLE 1:** E. coli strains used in the study

Name	Description	Source or reference
EDL933	Wild-type EHEC O157:H7	50
EDL933 Δ*eae*	EDL933 *eae* deletion mutant	51
EDL933 Δ*escN*	EDL933 *escN* deletion mutant	52
EDL933 Δ*espA*	EDL933 *espA* deletion mutant	53
EDL933 Δ*stx*	EDL933 *stx*_1_ *stx*_2_ deletion mutant	54
TUV93-0	Stx-negative derivative of EDL933	A. Donohue-Rolfe, Tufts University, USA
85-170	Stx-negative derivative of EHEC O157:H7 84-289	55
Walla-1	EHEC O157:H7	56
H0-7184-0336	EHEC O157:H7	G. Smith, Public Health England
E2348/69	EPEC O127:H6	57

### Cell culture and infection.

Human colon carcinoma T84 cells (ATCC CCL248) were cultured in Dulbecco's modified Eagle's medium/F-12 nutrient mixture supplemented with 10% fetal bovine serum (Sigma) and used between passages 49 and 65. Cells were seeded out in 24-well plates at a density of 10^5^ cells/well and grown for 7 days for full confluence. For Transwell experiments, 5 × 10^5^ cells/insert were seeded on collagen-coated Transwell filter inserts (diameter, 12 mm; pore size, 0.4 μm; Corning Costar). Transepithelial electrical resistance was monitored using an EVOM2 resistance meter with an STX2 electrode (World Precision Instruments), and values above 1,500 Ω · cm^2^ after 7 to 10 days of differentiation indicated establishment of epithelial barrier function. Confluent or polarized T84 cells were infected with approximately 2 × 10^7^ or 6 × 10^7^ bacteria, respectively, and incubated for the time periods indicated below. Medium was exchanged at regular intervals to prevent bacterial overgrowth and acidification. Cells were incubated at 37°C in a 5% CO_2_ atmosphere. At the end of the experiment, cells were washed twice in phosphate-buffered saline (PBS) to remove nonadherent bacteria and processed according to the need for further analysis.

### Quantification of adherent bacteria on polarized T84 cells.

Cell monolayers on filters were lysed in 1% Triton X-100 in PBS for 10 min. Serial dilutions of lysates were plated out on LB agar plates, and the numbers of CFU were determined after overnight incubation at 37°C.

### *In vitro* organ culture.

This study was performed with approval from the University of East Anglia Faculty of Medicine and Health Ethics Committee (reference 2010/11-030). All samples were provided through the Norwich Biorepository, which has NRES approval (reference 10/H0310/21). Biopsy samples from the terminal ileum or transverse colon were obtained with informed consent during colonoscopy of 14 adult patients (27 to 74 years old) and 2 pediatric patients (9 and 13 years old). Samples were taken from macroscopically normal areas, transported to the laboratory in IVOC medium, and processed within the next hour. IVOC was performed as described previously (21). Briefly, biopsy samples were mounted on foam supports in 12-well plates and incubated with 25 μl of a bacterial overnight culture (approximately 10^7^ bacteria). Samples were incubated in air–5% CO_2_ or 95% oxygen–5% CO_2_ at 37°C on a rocking platform for 8 h. At the end of the experiment, biopsy samples were washed twice in PBS to remove mucus and nonadherent bacteria and processed according to the need for further analysis.

### Scanning electron microscopy.

Samples were fixed with 2.5% glutaraldehyde in PBS and dehydrated through a graded acetone series. Specimens were dried using tetramethylsilane (Sigma), mounted on aluminum stubs, sputter coated with gold (Polaron SC7640 sputter coater; Quorum Technologies), and viewed with a JEOL JSM 4900 LV or Zeiss Supra 55 VP FEG scanning electron microscope. Bacterial adherence to biopsy sample epithelium was quantified by recording the presence or absence of adherent bacteria within approximately 250 fields of view of 50 by 35 μm^2^ covering the whole biopsy sample surface.

### Transmission electron microscopy.

Biopsy samples were fixed in 2.5% glutaraldehyde in 0.1 M PIPES [piperazine-*N*,*N*′-bis(2-ethanesulfonic acid)] buffer, postfixed in 1% aqueous osmium tetroxide, and dehydrated through a graded ethanol series. After embedding in LR White medium-grade resin, 1-μm semi-thin sections were cut with an ultramicrotome and stained with toluidine blue to locate adherent bacteria. Ultrathin sections (90 nm) were prepared from areas of interest, stained sequentially with uranyl acetate and lead citrate, and examined in an FEI Tecnai G2 20 Twin transmission electron microscope at 200 kV.

### Immunofluorescence staining.

Samples were fixed in 3.7% formaldehyde in PBS for 20 min and blocked/permeabilized with 0.1% Triton X-100 and 0.5% bovine serum albumin in PBS for 20 min. Samples were subsequently incubated in primary antibodies (goat anti-E. coli from Abcam; rabbit anti-EspA from Gad Frankel, Imperial College London; mouse anti-Tir from John Leong, Tufts University, USA) for 60 min, washed, and incubated in Alexa Fluor-conjugated secondary antibodies (Invitrogen) for 30 min. Filamentous actin was labeled with fluorescein isothiocyanate-conjugated phalloidin (Sigma). Samples were mounted in Vectashield medium (Vector Laboratories) and analyzed using a fluorescence light microscope (Axiovert 200M; Zeiss).

### Statistics.

All data are shown as means ± standard errors of the means (SEMs). Statistical analysis was performed using GraphPad Prism (version 5) software. Student's *t* test or one-way analysis of variance with Tukey's multiple-comparison test was used to determine differences between two or multiple groups, respectively. A *P* value of <0.05 was considered significant.

## RESULTS

### The EHEC adherence phenotype to T84 human colon carcinoma cells is dependent on polarization status.

**FIG 1:**
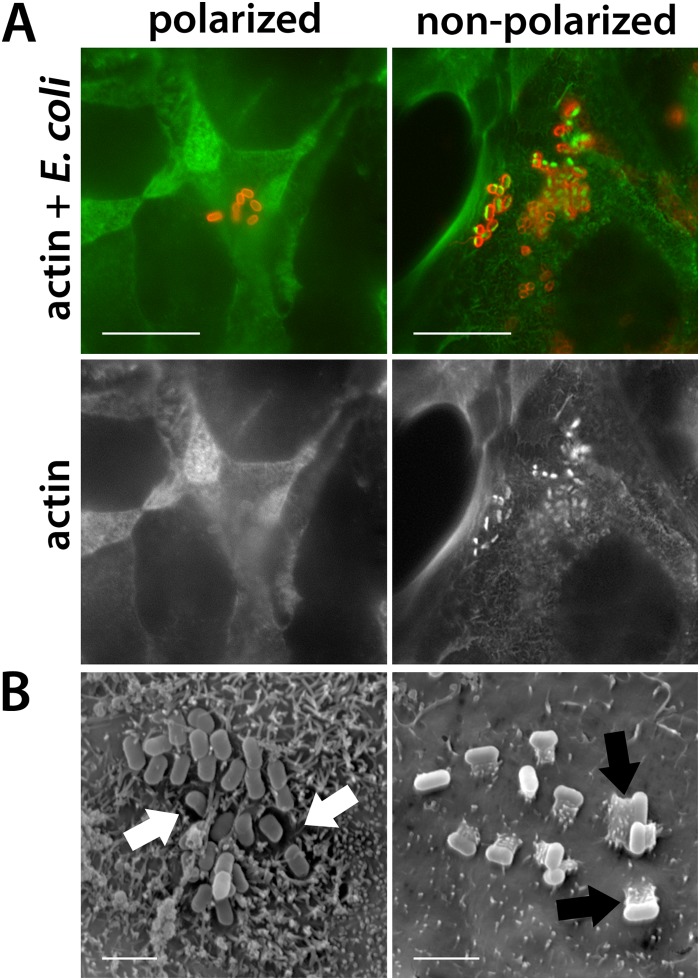
Different adherence phenotypes of EHEC on polarized and nonpolarized T84 cells. Confluent T84 cells on coverslips were infected with strain TUV93-0 for 5 h. Shown are representative images from two independent experiments performed in duplicate. (A) Immunofluorescence staining for actin (green) and E. coli (red). (Top) Merged images; (bottom) actin staining as a separate channel. Bars = 10 μm. (B) Scanning electron micrographs showing EHEC-associated microvillous effacement (white arrows) on polarized cells and actin pedestal formation (black arrows) on nonpolarized cells. Bars = 2 μm.

**FIG 2:**
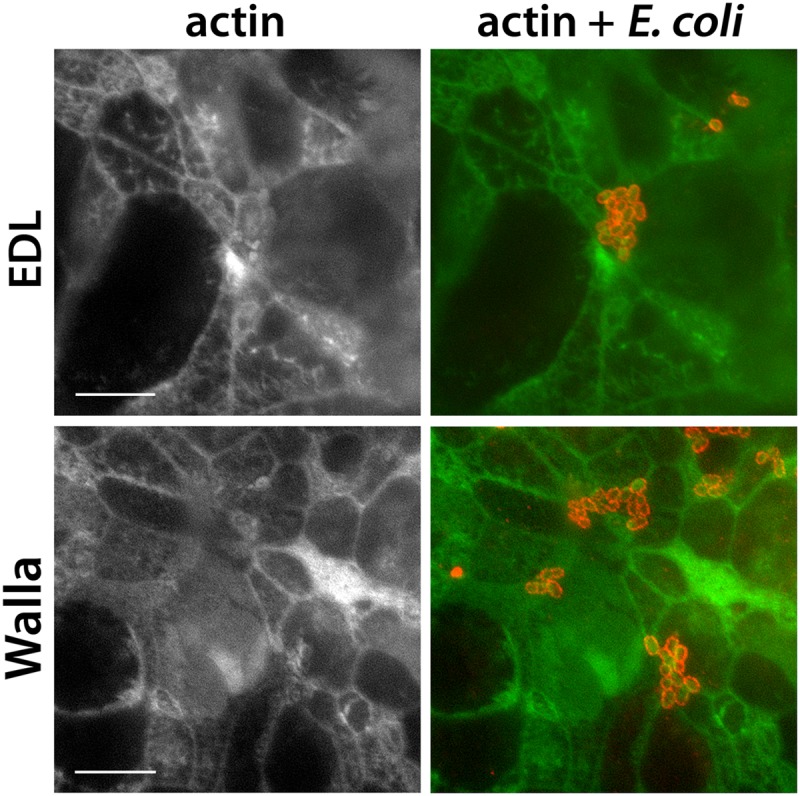
EHEC bacteria do not recruit actin in polarized T84 cells. T84 cells differentiated on Transwell membranes were infected with strain EDL933 or Walla-1 for 5 to 9 h. Immunofluorescence staining for actin (green) and E. coli (red). (Right) Merged images; (left) actin staining as a separate channel. Representative images after 9 h of infection from two independent experiments performed in duplicate. Bars = 5 μm.

To investigate the adherence of EHEC to T84 cells, confluent cell monolayers grown on coverslips were infected with Stx-negative strain TUV93-0 for 5 h, and the adherence phenotype was investigated by fluorescent actin staining and scanning electron microscopy. It was noted that T84 cells in the center of the monolayer showed signs of polarization, such as an actin-rich microvillous brush border, whereas cells at the margin of the coverslip appeared to be undifferentiated with few microvilli (Fig. 1). While EHEC bacteria adherent to marginal cells formed actin-rich pedestals, the bacteria on central polarized cells were not associated with polymerized actin but displayed signs of microvillous effacement (Fig. 1). Actin pedestal formation in polarized T84 cells was not impaired or obscured due to the high density of actin in the brush border, as T84 cells infected with the related A/E pathogen enteropathogenic E. coli (EPEC) showed actin recruitment on both polarized and nonpolarized cells (data not shown). Experiments were extended to EHEC wild-type strains EDL933 and Walla-1 using T84 cells grown on Transwell inserts. In this culture system, T84 cells reached full polarization status, as indicated by a high transepithelial electrical resistance, a column-shaped morphology, an actin-rich microvillous brush border, and the formation of tight junctions (data not shown). Infections were performed for 5 to 9 h, and no actin recruitment was observed for any of the strains tested (Fig. 2).

### EHEC colonizes human terminal ileal and colonic biopsy samples.

**FIG 3:**
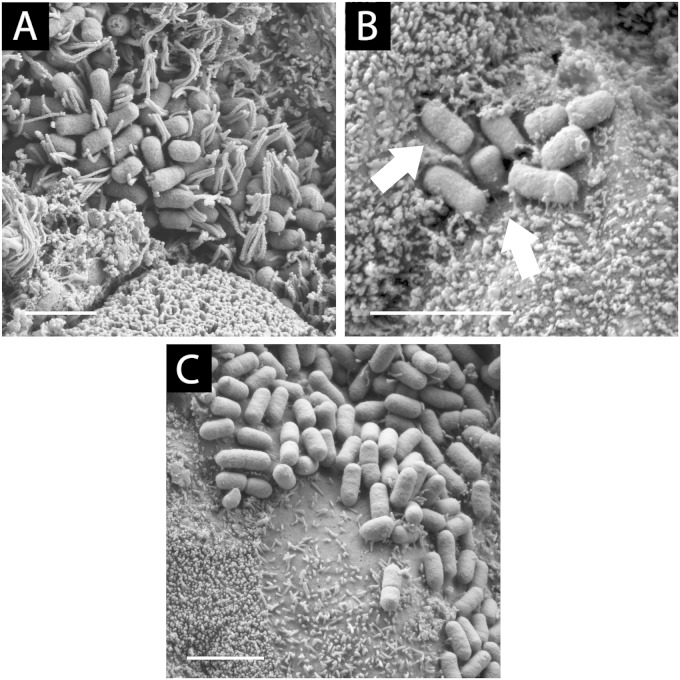
EHEC bacteria colonize human ileal and colonic biopsy samples. Endoscopic biopsy samples from the terminal ileum or transverse colon were infected with EDL933 for 8 h. (A) Scanning electron micrograph showing EHEC bacteria adhering to the terminal ileum and surrounded by elongated microvilli. (B) On the colon, a zone of microvillous effacement (arrows) was evident around adhering bacteria, and adjacent microvilli displayed a normal length. (C) An adherence phenotype similar to that shown in panel B was evident on pediatric colonic biopsy samples. Images are representative of those from three (A and B) and two (C) independent experiments performed in duplicate. Bars = 2 μm.

Colonization of human intestinal mucosa by wild-type EHEC was investigated by infecting terminal ileal and transverse colonic biopsy samples, taken from adults during routine endoscopy, with strains EDL933, Walla-1, or H0-7184-0336 for 8 h. Similar to T84 cell infections, IVOC was performed under atmospheric oxygen concentrations (20%). Scanning electron microscopy analysis revealed colonization of ileal and colonic biopsy samples by all strains (Fig. 3A and B; representative images are shown for EDL933). Similar to previous IVOC studies, extensive elongation of microvilli adjacent to adherent EHEC was observed on ileal biopsy samples (Fig. 3A). On colonic biopsy samples, microvillous effacement was apparent next to adhering bacteria, and surrounding microvilli displayed a normal length, which was similar to the phenotype observed on polarized T84 cells (Fig. 3B).

Colonic EHEC colonization has not been observed in previous IVOC studies using pediatric samples (17, 18). To examine whether young donor age was the reason for the lack of EHEC adherence, we also performed IVOC experiments using pediatric biopsy samples. Scanning electron microscopy analysis of EDL933-infected colonic biopsy samples demonstrated EHEC adherence similar to that in adult tissue samples (Fig. 3C).

### EHEC colonization of human colonic epithelium is not affected by Shiga toxin production.

**FIG 4:**
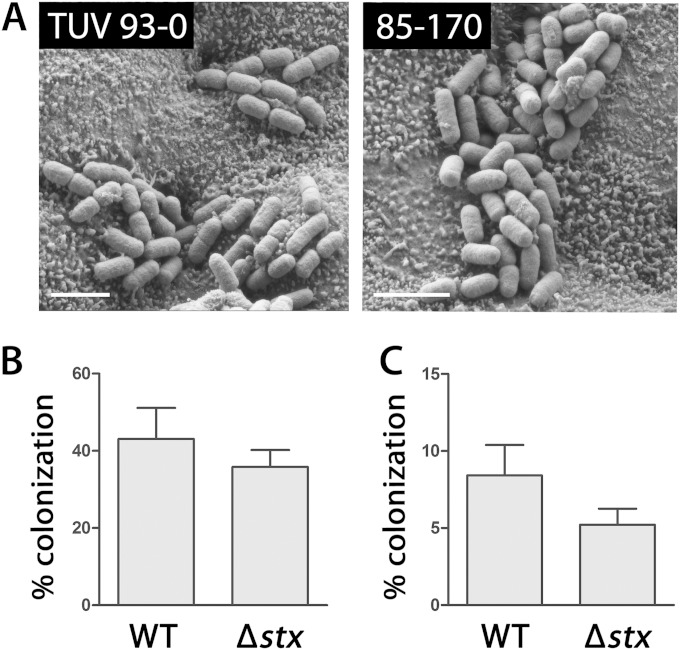
Colonization of colonic epithelium by EHEC is not affected by Stx production. (A) Scanning electron microscopy of biopsy samples from the transverse colon infected with Stx-negative strain TUV93-0 or 85-170 for 8 h. Images are representative of those from two independent experiments performed in duplicate. Bar = 2 μm. (B) Colonic biopsy samples were infected with wild-type (WT) EDL933 or an isogenic Stx deletion mutant (Δ*stx*) for 8 h. Samples were viewed by scanning electron microscopy, and epithelial colonization was quantified by recording the presence or absence of adherent bacteria in approximately 250 fields of view. Colonization is expressed as the percentage of the fields of view containing adherent bacteria. Data are shown as means ± SEMs from two independent experiments performed in triplicate. (C) Polarized T84 cells were infected with wild-type EDL933 or EDL933 Δ*stx* for 6 h. The numbers of adherent bacteria were quantified by plating serial dilutions of cell lysates and determining the numbers of CFU. Colonization is expressed as the percentage of adherent bacteria relative to the inoculum. Data are shown as means ± SEMs from five independent experiments performed in duplicate.

Previous IVOC studies on Stx-negative EHEC have failed to show the direct colonization of colonic biopsy samples (17, 18). As Stxs have been implicated in EHEC adherence to human epithelial cells and colonization of mouse intestine (19), we investigated whether Stx production was required for colonic binding. IVOC of colonic biopsy samples with Stx-negative strains TUV93-0 and 85-170 (used in previous studies) was performed. As shown in Fig. 4A, both strains showed good colonic colonization with a phenotype similar to that of wild-type EHEC strains.

In addition, adherence of EDL933 and an isogenic Stx-deletion mutant to colonic biopsy samples and polarized T84 cells was quantified. Figures 4B and C show that there was no significant difference in the number of cell-associated bacteria between the two strains (*P* = 0.24 and *P* = 0.1236, respectively).

### Involvement of EHEC T3S in colonic adherence.

**FIG 5:**
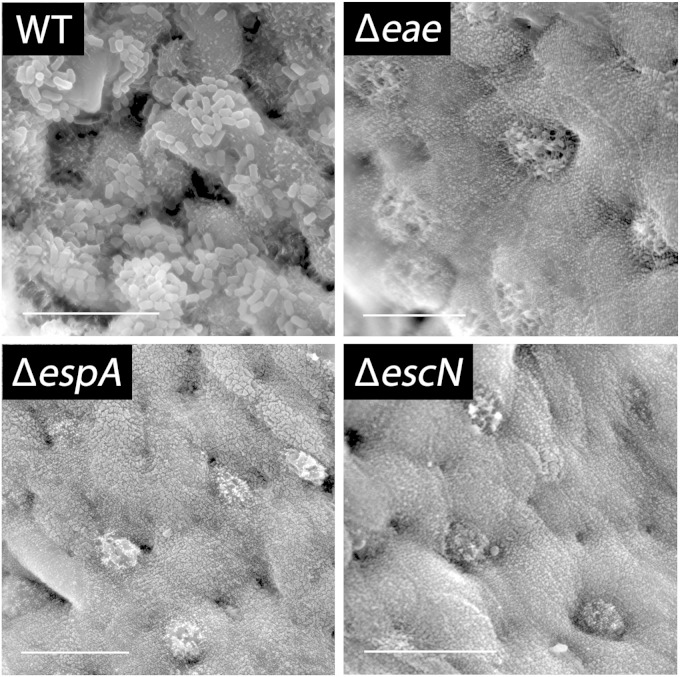
EHEC colonization of colonic biopsy samples is dependent on intimin and T3S. Scanning electron micrographs of biopsy samples from the transverse colon infected with wild-type (WT) EDL933 or isogenic EspA, EscN, or intimin (*eae*) mutants for 8 h. Images are representative of those from four independent experiments performed in duplicate. Bars = 10 μm.

**FIG 6:**
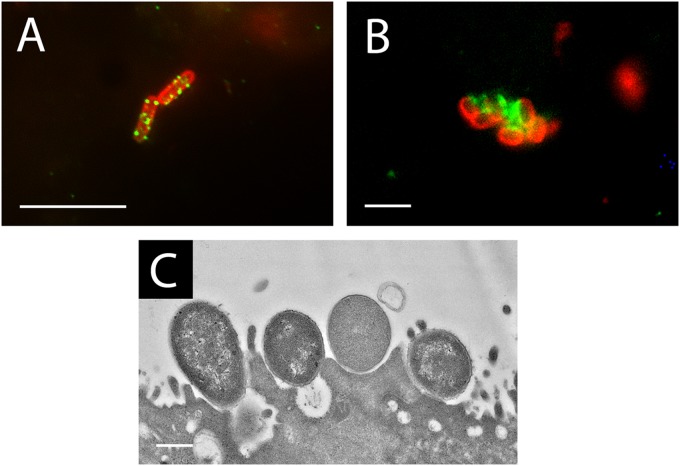
EHEC bacteria form typical A/E lesions on human colonic biopsy samples. Colonic biopsy samples were infected with EDL933 for 8 h. Immunofluorescence staining was performed for EspA (A) or Tir (B) in green and E. coli in red. (C) Transmission electron micrograph showing intimate EHEC adherence to host cell membrane and loss of microvilli. Images are representative of those from two independent experiments performed in duplicate. Bars = 2 μm (A, B) or 0.5 μm (C).

**FIG 7:**
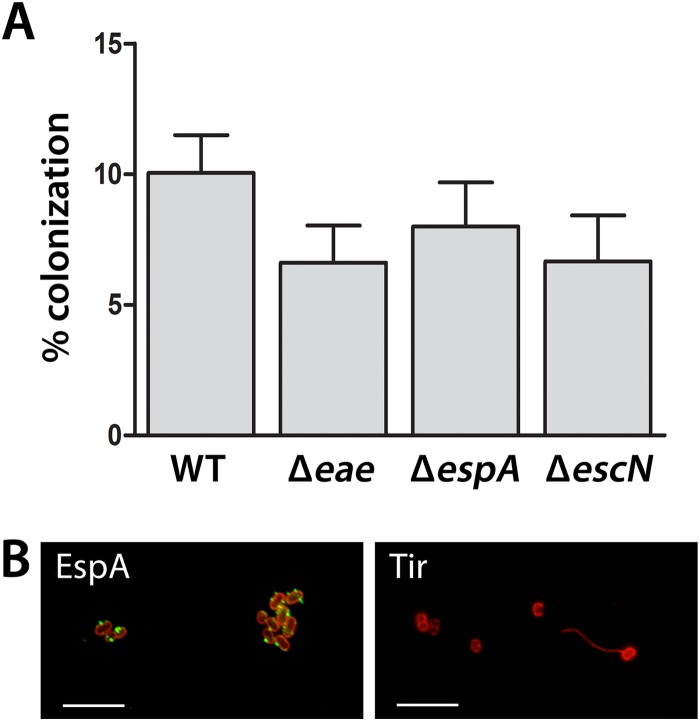
Adherence of EHEC to polarized T84 cells is independent of intimin and T3S and does not involve Tir translocation. (A) Polarized T84 cells were infected with wild-type (WT) EDL933 or isogenic EspA, EscN, or intimin (*eae*) mutants for 6 h. The numbers of adherent bacteria were quantified by plating serial dilutions of cell lysates and determining the number of CFU. Colonization is expressed as the percentage of adherent bacteria relative to the inoculum. Data are shown as means ± SEMs from four independent experiments performed in duplicate. (B) Immunofluorescence staining of polarized T84 cells infected with EDL933 for 6 h. Green, EspA and Tir; red, E. coli. Images are representative of those from two independent experiments performed in duplicate. Bars = 5 μm.

We next determined whether EHEC adherence to colonic epithelium was dependent on T3S or intimin. IVOC of colonic biopsy samples was performed using EDL933 mutants deficient in EspA (the translocation filament), EscN (the cytoplasmic ATPase of the T3SS), or intimin, and colonization was evaluated by scanning electron microscopy. As shown in Fig. 5, all mutants failed to colonize, whereas the wild type showed good adherence. Quantification of colonized sample areas yielded 25.99% ± 7.19% for the wild type, whereas no areas with adherent bacteria (0%) were detected for any of the mutant strains. Immunofluorescence staining and transmission electron microscopy were subsequently used to evaluate A/E lesion formation. As shown in Fig. 6, adherent EHEC bacteria were associated with EspA filaments and translocated Tir and demonstrated intimate attachment and microvillous effacement. In contrast, adherence of EDL933 to polarized T84 cells was not significantly affected by the absence of EspA, EscN, or intimin (Fig. 7A). Immunofluorescence staining of EDL933-infected polarized T84 cells demonstrated the formation of EspA filaments, but translocated Tir was absent in monolayer-associated cells (Fig. 7B) and detected only in detaching cells which had lost cell polarity (data not shown).

### High levels of oxygen suppress EHEC adherence and A/E lesion formation on human colonic biopsy samples.

**FIG 8:**
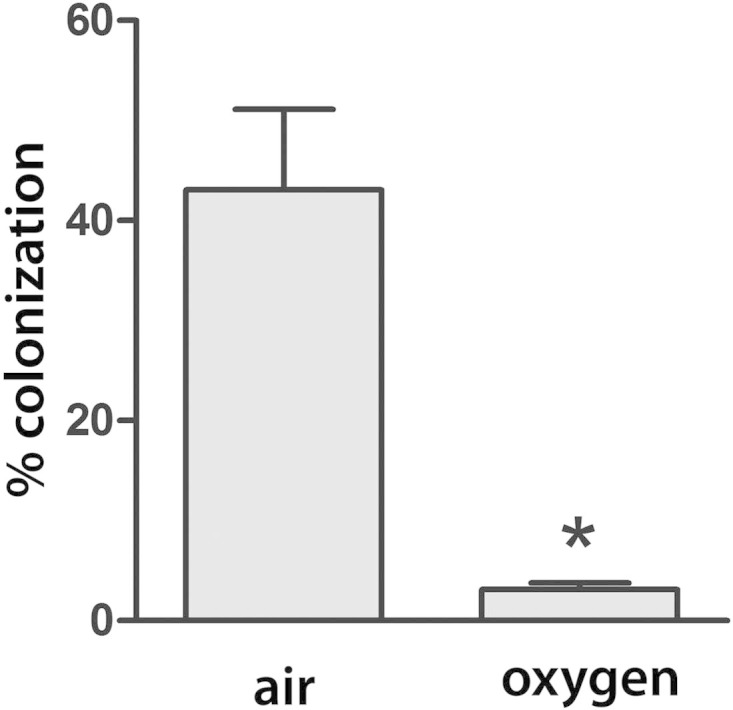
EHEC A/E lesion formation on colonic biopsy samples is suppressed by oxygen-rich conditions. IVOC of colonic biopsy samples with EDL933 was performed for 8 h under high (oxygen) or atmospheric (air) oxygen levels. Samples were viewed by scanning electron microscopy, and epithelial colonization was quantified by recording the presence or absence of adherent bacteria in approximately 250 fields of view. Colonization is expressed as the percentage of fields of view containing adherent bacteria. Data are shown as means ± SEMs from two independent experiments performed in triplicate. *, *P* < 0.05.

Our previous studies have demonstrated inhibition of EHEC T3S and A/E lesion formation on polarized T84 cells by oxygen (20). To investigate whether oxygen also affected EHEC A/E lesion formation on colonic biopsy samples and might explain the lack of colonization observed in previous studies (17, 18), IVOC was performed under high (95%, as in previous studies [17, 18]) or atmospheric (20%, as in this study) oxygen levels. As shown in Fig. 8, colonization of EDL933 was significantly inhibited under oxygen-rich conditions.

We also performed IVOC under microaerobic (∼1.5% oxygen) conditions similar to those in the environment in the human colon but observed severe epithelial cell extrusion even on noninfected samples after 5 h of incubation (data not shown).

## DISCUSSION

EHEC is considered a colonic pathogen, and the clinical histopathology of HC is predominantly observed in the ascending and transverse colon (1, 22). However, the intestinal pathogenesis of EHEC has not been well explored, and this is partly due to the lack of suitable animal model systems. Major obstacles include the failure of EHEC to efficiently colonize the mouse or rabbit intestinal tract without prior removal of the resident microflora (23) and the expression of the Stx receptor Gb3 by mouse and rabbit intestinal epithelium, in contrast to the situation in humans (24–26). Therefore, cell culture models have been widely applied, and the T84 human colon carcinoma cell line has been used in many EHEC studies, as it has the structural characteristics of colonic crypt cells (27) and, like human intestinal epithelium, does not express significant amounts of Gb3 and is resistant to Stx cytotoxicity (26).

In our study, we have found that EHEC bacteria adhering to polarized T84 cells do not form typical A/E lesions. While formation of the EspA filament and microvillous effacement were evident, no Tir translocation or actin polymerization was detected in association with adherent bacteria. In addition, EHEC colonization was not significantly affected by the absence of EspA, intimin, or T3S, which suggests the involvement of other adherence factors, such as fimbriae, autotransporters, or flagella (28). These findings are consistent with those of previous studies, which have failed to detect EHEC actin pedestals in confluent T84 cells (29, 30). Interestingly, EHEC bacteria were still able to modulate host cell signal transduction and function, such as intracellular calcium levels, epithelial barrier function, and ion transport, which suggests that T3S into polarized T84 cells can occur independently of the intimin-Tir interaction or actin polymerization (29–32).

It is currently unknown which bacterial factors cause microvillous effacement during EHEC infection, but findings on the related A/E pathogen EPEC appear to be dependent on the model system used, with adherence phenotypes even differing between Caco-2 cell subclones (33). Whereas microvillous effacement and bacterial sinking in Caco-2 cells have been reported to be dependent on intimin and Tir (34), EPEC mutants with mutations in intimin or Tir still cause microvillous effacement and effacing footprints in pediatric duodenal IVOC (35). On the other hand, EPEC microvillous effacement in porcine ileal IVOC appears to be intimin dependent but independent of Tir (36). Our study on polarized T84 cells demonstrates that EHEC effacement can occur independently of Tir translocation into the host cell membrane.

A different EHEC adherence phenotype was apparent on nonpolarized T84 cells at monolayer margins or on detaching cells, where translocated Tir and actin pedestals were observed. This could be due to the availability of phosphatidylethanolamine or other receptors for EHEC binding which become exposed on the cell surface after apoptosis or cell shedding (37, 38). The ability of EPEC to form actin pedestals on polarized T84 cells suggests that this pathogen uses different receptors for initial binding than EHEC and that these receptors are readily expressed on the apical cell membrane. Another possibility for the failure of EHEC to form actin pedestals on polarized T84 cells might be related to particular properties of the apical T84 cell membrane which would prevent proper EHEC Tir insertion or clustering by intimin.

Despite the presence of colonic pathology, it has been controversial whether EHEC can colonize human colonic epithelium *in vivo*, as adherent bacteria have not been reported during clinical infections (15, 39). It has been argued that this may be because of the progressed stage of disease at the time of endoscopy, when bacterial adhesion may have diminished or be difficult to identify due to extensive tissue damage (1, 17). In contrast, EHEC infections in gnotobiotic piglets, infant rabbits, and neonatal calves have shown colonization of the terminal ileum, cecum, and colon (12–14, 40). Adherent bacteria were associated with characteristic A/E lesions accompanied by intimate attachment and loss of microvilli, and adherence was dependent on intimin-Tir interaction (12, 13, 41). Ileal and colonic A/E lesions have also been reproduced in bovine intestinal IVOC and shown to be dependent on Tir (42). In contrast, human IVOC studies using pediatric biopsy samples have demonstrated EHEC binding and A/E lesion formation on terminal ileum but not colon (17, 18). However, some minimal nonintimate adherence to colonic explants was observed after previous incubation of EHEC with terminal ileal biopsy samples (17). These findings have led to the hypothesis that EHEC initially colonizes the terminal ileum and Peyer's patches, where bacteria are primed for subsequent spread and infection of the colon. Similar colonization dynamics have been described for the mouse A/E pathogen Citrobacter rodentium, which demonstrates primary adherence to the lymphoid cecal patch before establishing colonization of the colon (43). Interestingly, a recent study using human intestinal xenografts in mice has reported T3S-dependent EHEC A/E lesion formation on human colon but not on small intestine (44).

In our study, we have found EHEC colonization of human terminal ileum and colon *ex vivo*. Typical A/E lesions similar to those previously described on terminal ileal biopsy samples were observed on colonic explants, demonstrating intimate attachment, microvillous effacement, and Tir translocation beneath adherent bacteria (18, 45). Interestingly, colonic A/E lesions were not accompanied by elongation of the surrounding microvilli, as observed on terminal ileum. This has also been observed on bovine IVOC and human intestinal xenografts and might reflect differences in the organization of the brush border cytoskeleton in the small intestine and colon (42, 44). Similar to previous human intestinal xenograft and animal studies, A/E lesion formation on human colonic explants was dependent on T3S and intimin (13, 41, 44).

In addition to Tir, the host cell protein nucleolin has been described to be a host receptor for intimin (46), and previous studies have shown that Stxs enhance EHEC adherence to HeLa cells and intestinal colonization of mice by inducing surface expression of nucleolin (19). As former human IVOC studies have been performed with Stx-negative EHEC strains (17, 18), we investigated whether Stxs could promote colonic adhesion. Our findings on Stx-negative mutants showed that Stx production did not significantly affect EHEC adherence to human colonic epithelium, which agrees with previous results in infant rabbits, where Stx expression did not alter colonization levels (13).

Other differences from earlier human IVOC studies by Phillips and colleagues (17, 18) which might explain the discrepancies in colonic colonization include the use of adult versus pediatric biopsy samples and lower oxygen concentrations during IVOC. Whereas the influence of age on EHEC colonic infection has not been investigated, IVOC studies with EPEC have demonstrated no significant difference in EPEC binding to adult versus pediatric biopsy samples (47). As we also observed EHEC colonization of pediatric colonic biopsy samples, age was not the determining factor for our findings.

In contrast, we found that the high oxygen levels (95%) commonly used in IVOC to ensure sufficient tissue oxygenation and survival (21, 48) suppressed EHEC adherence to colonic biopsy samples. This is in agreement with the findings of our previous study demonstrating that lower oxygen levels promote EHEC adherence and T3S on polarized T84 cells (20). Similar results have been reported for bovine intestinal IVOC, where the EHEC colonization observed with air (20% oxygen) was improved compared with that achieved with 95% oxygen without compromising tissue integrity (42). Lower oxygen levels are also likely to explain EHEC A/E lesion formation in human colonic xenografts (44). Interestingly, high oxygen levels did not abolish EHEC A/E lesion formation on terminal ileal biopsy samples (17, 18), suggesting higher levels of adherence to the small intestine than to the colon. This might be related to a thinner mucus layer with less microbiota and easier access to the epithelium (49).

In summary, our study demonstrates for the first time that EHEC forms typical A/E lesions on human colon *ex vivo* which are dependent on T3S and intimin. Importantly, A/E lesion formation is dependent on oxygen levels and suppressed by the oxygen-rich culture conditions generally used in IVOC. In contrast, adherence to polarized T84 cells is mediated by factors other than EspA and intimin and does not involve Tir translocation into the host cell membrane. This study emphasizes the difference between cell culture experiments and more relevant model systems, such as IVOC, and suggests that during human infection EHEC forms stable A/E lesions which are likely to contribute to colonic pathology.
